# Investigating Dissolved Glucose as an Alternative Nutrient Source for Bivalve Larvae

**DOI:** 10.1155/anu/5203885

**Published:** 2025-11-20

**Authors:** Andy Jordan, Kim Thompson, Andrew Jeffs

**Affiliations:** ^1^Institute of Marine Science, University of Auckland, Auckland, New Zealand; ^2^Te Huata Mussel Hatchery Ltd., Te Rūnanga O Te Whānau, Te Kaha, New Zealand; ^3^School of Biological Sciences, University of Auckland, Auckland, New Zealand

**Keywords:** bivalve, glucose, hatchery culture, larvae, nutrient uptake

## Abstract

The production of live microalgae represents a major cost in the operation of bivalve hatcheries, as it is the primary food used for larval rearing. This study investigated whether dissolved glucose could reduce the reliance on live microalgae as a sole feed input without negatively affecting bivalve larval performance during the rearing of Greenshell™ mussels (*Perna canaliculus*). Larvae from a single spawning cohort were raised to Day 10 post-fertilisation and then split into two feeding treatments: (1) control, fed only live microalgae in a continuous flow-through system and (2) glucose treatment, fed live microalgae in a continuous flow-through system, which was interrupted for 2 h, daily during which 10 μg mL^−1^ of glucose dissolved in seawater, with a 20 min tank flush before and after exposure. The larvae were raised to settlement, during which time growth, microalgae consumption, losses of larvae at screening, settlement success and stable isotope composition (*δ*^13^C and *δ*^15^N) were assessed. Under these experimental conditions, substituting microalgae with glucose for 2 h daily did not alter the performance or isotopic composition of the larvae despite substituting an estimated 8.3% of live feed. This result provides a foundation to further test and refine the delivery of soluble nutrients, like glucose, as a means to reduce operational costs in bivalve hatcheries.

## 1. Background

In New Zealand, the Greenshell mussel (*Perna canaliculus*) is the predominant aquaculture species, generating a total annual revenue of NZ$347 M in 2023, which accounts for more than half of the country's total aquaculture revenue [1]. However, approximately 80% of the spat used by the industry is wild-caught, washing ashore on Te Oneroa-a-Tōhē (Ninety Mile Beach) attached to drift seaweed [2, 3]. The remaining spat is obtained from spat-catching rope and a limited number from hatcheries [4]. The industry's reliance on wild-sourced spat leaves New Zealand farmers waiting for spatfall to seed out new lines during farming cycles, which is inconsistent and seasonal [5]. Additionally, these wild spat often experience anoxic stress and prolonged periods without food in the turbulent surf zone before being transferred to farms, leaving them in poor physiological condition by the time they are seeded out [6, 7].

The hatchery production of mussel spat is a more reliable source compared to wild-caught spat, as they can be supplied all year round and are far less variable in terms of their condition, size and genetics, which are all believed to impact their subsequent performance on farms [8–10]. Despite these advantages, the expansion of hatchery production of Greenshell mussel spat is hindered by the high capital and operational costs for hatcheries. Bivalve hatcheries are highly dependent on high-quality live unicellular microalgae as feed for the larvae and spat. The production of live microalgae feed for larvae and juvenile bivalves typically accounts for between 30% and 40% of total operational expenses for a hatchery producing spat [11, 12]. Consequently, more cost-effective alternative feeds to microalgae need to be developed to reduce the hatchery operational costs and, in so doing, help to facilitate the efficiency of hatchery production of mussel spat in New Zealand.

The primary advancement in larval feeding since the establishment of bivalve hatchery culture is the improved performance of larvae when fed multispecies live microalgae diets [13, 14]. Multispecies diets enhance larval growth and survival compared to single-species diets because the precise nutritional requirements of larvae are difficult to ascertain, and the proximate composition of microalgae varies considerably, even when cultured under the same culture conditions [15]. There have been ongoing efforts for many years to produce alternative feeds to live microalgae in an effort to reduce operational costs, with research primarily focused on the development of microencapsulated [16], freeze-dried [17] or spray-dried microalgae [18]. These alternatives have typically shown promise in experimental trials, successfully rearing larvae to metamorphosis. However, their commercial application is challenging, as feed particles need to be precisely between 0.2 and 30 μm in diameter for larval ingestion during early development. However, particles with a diameter of 2–4 μm are most effective for ingestion [19–22]. Furthermore, preserved microalgal products generally have inferior nutritional composition compared to live microalgae, resulting in decreased larval performance [22].

One strategy for reducing feeding costs in bivalve hatcheries that has not yet been explored is using dissolved organic material (DOM) to partially replace live microalgae. Bivalves absorb DOM from seawater using the gills or the velum in the case of larvae, where it is actively transported across cell membranes [23]. Bivalve larvae have been shown to absorb and utilise dissolved amino acids, carbohydrates and vitamins from seawater [24–28]. For example, the larvae of Pacific oysters (*Magallana gigas*) can be grown solely on dissolved amino acids, glucose and fat-soluble vitamins under axenic conditions, indicating that dissolved organic matter can play a significant role in meeting the overall nutritional requirements of this species [25]. Despite these early findings, there has been little or no further exploration of how dissolved organic matter could be used to reduce some of the costs associated with larval rearing in bivalve hatcheries.

The early stages of bivalve larval development are characterised by intense morphogenetic activity fuelled by stored energy provisioned to the eggs from the adult [29]. During subsequent larval development until settlement, high levels of exogenous nutrient sources are typically required to maintain the development [30]. Lipids, particularly the omega-3 fatty acids eicosapentaenoic acid (EPA, 20:5*n*-−3) and docosahexaenoic acid (DHA, 22:6n–3) have been identified as key dietary components during this period due to their role in maintaining membrane structure and function [31, 32]. Carbohydrates have often been overlooked in larval nutrition, as they make up a small proportion (i.e., <3%) of larval tissues [33, 34]. Despite contributing only a small percentage of organic matter, there is evidence that carbohydrates play a critical role in energy production and somatic development, and consequently their supply affects larval growth and survival [35]. For example, the carbohydrate content of blacklip pearl oyster (*Pinctada margaritifera*) larvae increased 50% between Days 18 and 21 of rearing. Therefore, while carbohydrates may constitute a small biochemical component of larvae relative to protein and lipids, they appear to be an important dietary component for larvae prior to metamorphosis.

Little is known about the nutritional requirements of Greenshell mussel larvae, with hatchery feeding regimes similar to those used for many other bivalve species, with mixed microalgae diets generally consisting of two diatom species and a flagellate species [8, 13, 36]. Specifically, the microalgae species *Chaetoceros calcitrans* and *Tisochrysis lutea* are effective in promoting higher growth and survival, most likely due to the higher content of protein and DHA and EPA [8, 36, 37]. Much less is known of the role of carbohydrates in the development of Greenshell mussel larvae. However, following metamorphosis, carbohydrates become the primary source of energy for spat and serve as a useful indicator of overall nutritional condition [10, 38–40]. Many carbohydrates, such as glucose, fructose and sucrose, are readily available at relatively low cost, are highly soluble in seawater and can be absorbed by larvae directly from seawater [28]. Supplementing microalgae diets with dissolved glucose can substantially enhance spat growth, increasing performance 2.7 times compared to spat fed only microalgae [38]. However, it remains unknown if Greenshell mussel larvae possess the same capacity to rapidly metabolise dissolved sugars and if this can provide an alternative source of nutrition during hatchery rearing. Addressing this knowledge gap is important for determining whether short daily exposures to dissolved nutrients could expand the range of feeding strategies available in larval culture and contribute new insights into the nutritional physiology of bivalve larvae.

The aim of this study was to investigate if dissolved glucose could be used as a partial replacement for feeding live microalgae in the larval rearing of Greenshell mussels.

## 2. Materials and Methods

### 2.1. Larvae Source

Broodstock Greenshell mussels were conditioned in a semi-recirculating system where wild microalgae were delivered via an artificially fertilised saltwater pond at the Te Huata Ltd. hatchery. Spawning of broodstock was induced by thermal shock, and the initial gamete release allowed individuals to be sexed and then isolated. Egg density was stocked at 1000 mL^−1^, and sperm was added at a ratio of 500 egg^−1^. Following fertilisation, the embryos were incubated for approximately 48 h, until the embryos had transformed into early D veligers.

### 2.2. Larval Rearing System

The D veligers were transferred into a Cawthron Ultra-Density Larval rearing (CUDL) system [41], and previously described in Ragg et al. [8]. The system consisted of 12 bullet-shaped 2.5 L acrylic tanks, each receiving seawater from a ø 5 mm glass tube at a rate of 80 mL^−1^ h^−1^ for the first 8 days and then 140 mL^−1^ h^−1^ for the remainder of the experiment.

Incoming seawater was supplied to the tanks via a manifold system and filtered to 1 μm following sequential treatments through a sand filter, a 5 μm bag filter and a 1 μm cartridge filter. Water quality was maintained within the following ranges: dissolved oxygen 100%–107% saturation, ammonia ≤ 0.015 mg L^−1^ NH_3_-N, pH 7.78–8.50 and temperature 17 ± 1°C. Continuous flow ensured that the water in each tank was fully exchanged approximately twice per hour. Every 2 days, tanks were drained through screens with progressively larger mesh sizes, with the selected mesh size designed to retain approximately 90% of the larvae and eliminate slow-growing individuals.

On Day 10, the larvae from all tanks were pooled together and split evenly into 12 CUDLS. Two feeding treatments (control and glucose) were randomly assigned to six tanks each.

The larval culture continued for a further 10 days, with settlement cues identified by individuals developing into ready-to-settle pediveligers characterised by the presence of eye spots and an exploratory foot. At day 20 these pediveligers were separated using a 175 μm screen and transferred back to their tanks with 5 m of presoaked coir rope suspended in coils within the tank to provide sufficient settlement substrate to accommodate an estimated potential density of 5000 settled pediveligers m^−1^ of coir rope. The tanks were put on static culture with seawater changes performed each morning for the next 5 days, while the pediveligers settled onto the coir. Aeration in each tank was provided through a vertical glass tube that delivered air to the base of the tank.

### 2.3. Feeding Treatments

The two dietary treatments, each with six replicate tanks of cultured Greenshell mussel larvae, consisted of (1) a mixed microalgae diet delivered continuously and (2) a mixed microalgae diet delivered continuously for 21.3 h daily, followed by a 20 min seawater flush, 2 h of seawater containing 10 μg mL ^−1^ dissolved glucose (Sigma Aldrich), a second 20 min seawater flush and then a return to standard microalgae feeding. Larvae were cultured under a continuous 24 h photoperiod, with glucose incubations conducted each day at approximately 12:00 PM 10 µg mL^−1^ of glucose, delivered over a 2 h incubation, was selected because it was the lowest concentration shown to be nutritionally beneficial for Greenshell mussel spat [38, 39], while also minimising the risk of bacterial proliferation in CUDL's during exposure.

The mixed microalgae diet consisted of *Chaetoceros calcitrans*, *Chaetoceros muelleri* and *Tisochrysis lutea*. The ratio of these microalgae species was adjusted throughout larval development. Initially, only *C. calcitrans* was provided, as early-stage larvae can feed successfully on this species. As larvae grew and became capable of feeding on a wider range of particles, *T. lutea* and then *C. muelleri* were introduced, resulting in a mixed diet of all three species by the end of the experiment. This approach aligns with standard larval rearing protocols, as mixed-species diets improve larval nutritional condition and settlement [8, 13, 36].

The concentration of microalgae in the flow-through system was maintained at 40 cells μL^−1^ from day 0 until Day 8, after which it increased to 60 cells μL^−1^, as these concentrations have been shown to be optimal for larval growth and survival [8]. Microalgae cell concentrations were measured using fluorescence readings which were calibrated to corresponding cell densities (Cyclops-7, Turner Designs, Sunnyvale, CA, USA).

During the 5-day pediveliger settlement stage of the experiment, the mussels were fed the mixed microalgae diet (*C. calcitrans*, *C. muelleri* and *T. lutea*), which was administered manually once daily to every tank at a concentration of 40 cells μL^−1^.

### 2.4. Larval Performance

#### 2.4.1. Growth

Measurements of larval prodissoconch length in each tank were taken every 2 days by taking a 1 mL random sample of well-mixed culture water containing larvae from each tank and fixing in 0.3% formalin in seawater and then measuring 50 larvae under a microscope (Olympus DP74 camera mounted on a CKX53 inverted microscope, ×40 magnification) by taking the longest distance across the prodissoconch, that is, the prodissoconch length.

#### 2.4.2. Screen Losses and Daily Observations

Counts of larvae in each tank were done every 2 days after screening so that larval losses from each tank between each screening event could be estimated. Counts were performed by taking three random subsamples of 200 µL of seawater containing larvae from each tank and counting the larvae under a microscope, then multiplying up the mean count by the measured volume of the tank, that is, 2.5 L.

#### 2.4.3. Settlement Success

The total number of pediveliger larvae that successfully settled in each tank (i.e., spat yield) 5 days after reaching ready-to-settle pediveligers was determined by taking a 5 cm section from the bottom, middle and top of the coir rope from each tank and counting the number of settled plantigrades. The counts of settled mussels on the three 5 cm subsamples of coir were used to estimate the mean density of plantigrades m^−1^ of coir rope in each tank. Settlement success was calculated by dividing the total number of plantigrades settled on the coir rope by the total number of pediveligers in the tank at the time of the coir rope being added to the tank.

#### 2.4.4. Microalgae Cell Consumption

The feeding activity of the larvae in each tank was determined daily 3 h after glucose exposure by measuring the difference in the concentration of microalgae cells in the seawater coming into each tank versus that leaving the tank, including a correction provided by using a seawater blank without microalgae, following a similar methodology outlined in Ragg et al. [8].

  $$\begin{array}{c} \text{Consumption}=\left(A_{\text{in}}-A_{\text{out}}\right)-\Delta_{\text{blank}}. \end{array}$$

### 2.5. Analysis of Stable Isotopic Composition (*δ*^13^C and *δ*^15^N)

Stable isotope analysis was used to assess potential differences in the nutritional condition of settled plantigrades due to the small quantity of biomass available from the experiment. Plantigrades from each replicate tank were washed off the coir rope and separated from the rope debris, washed in deionised water and then lypholised for 24 h. Lypholised spat (~0.45 mg) were then loaded into tin capsules for stable isotope analysis. Stable isotope analysis of carbon (*δ*^13^C) and nitrogen (*δ*^15^N) was conducted using a Eurovector (EuroEA 300) elemental analyser coupled to an isoprime IRMS continuous flow mass spectrometer. Leucine was used as a drift standard and for linearity checks after every 10 samples, with other internal standards (cane sugar, beet sugar for ^13^C; EDTA, caffeine and IAEA N1/N2 for ^15^N) run at the beginning and throughout the sequence to ensure consistency. Corrections for linearity and signal 'stretch' were applied using these standards, and the instrument was calibrated to maintain an accuracy of ± 0.2‰ for *δ*^13^C and ± 0.3‰ for *δ*^15^N.

### 2.6. Statistical Analyses

A linear mixed-effects model was fitted to the measurements of prodissoconch length, taken on Day 10 (when glucose feeding began) and then subsequently every 2 days. The prodissoconch length was predicted by the fixed effects of time and day, with replicate tanks included in the model as a random effect. The slope of the regression from the mixed-effects model was used to determine daily growth for both the control and glucose treatments. To compare sizes at the outset of the experiment (Day 10) and the end of the experiment (Day 20), post hoc pairwise comparisons were applied to the estimated marginal means (EMMs) from the mixed-effects model. The effects of treatment and day on larval screening loss and microalgae consumption were analysed using a two-way analysis of variance (ANOVA). Treatment and day were included as fixed factors, and their interaction was assessed to determine whether treatment effects varied overtime. For settlement success and stable isotope composition (*δ*^13^C and *δ*^15^N), differences between the two treatments at the end of the experiment were assessed using Welch's two-sample *t*-tests, which account for unequal variances between groups. All data were checked for normality and homogeneity of variances, and where data violated these parametric assumptions, they were log-transformed and reassessed to confirm conformity. Percentage screening losses were transformed using an arcsine transformation prior to being subject to an ANOVA. Where significant main effects were identified by ANOVA, pairwise Tukey HSD post hoc tests were used to identify differences among pairs of individual means.

## 3. Results

### 3.1. Larval Growth

**Figure 1:**
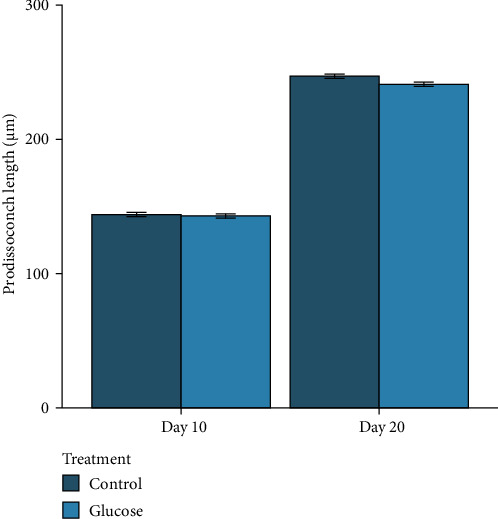
The mean prodissoconch length of larvae in the control and glucose treatments at the outset of the experiment (Day 10) and at Day 20 when they had reached pediveligers.

The mean prodissoconch length of the mussel larvae at the beginning of the feeding experiment (Day 10) was 144.0 µm (±1.6 SE) in the control and 143.0 µm (±1.6 SE) in the glucose treatment and were not different (*t*_(3583)_ = 0.60, *p* = 1.00, Figure 1). At Day 20 the mean prodissoconch length of the ready-to-settle pediveligers in the control was 247.0 µm (±1.6 SE) and 241.0 µm (±1.6 SE) in the glucose treatment and were not different (*t*_(3583)_ = 3.07, *p* = 0.09). Additionally, there was no difference in the mean daily growth of larvae between the control and glucose treatments from Day 10 to 20 (*t*_(3583)_ = −1.75, *p* = 0.08), with daily larvae growth in the control treatment 10.3 μm day^−1^ (±0.2 SE) versus 9.9 μm day^−1^ (±0.3 SE) in the glucose treatment.

### 3.2. Screening Losses

**Figure 2:**
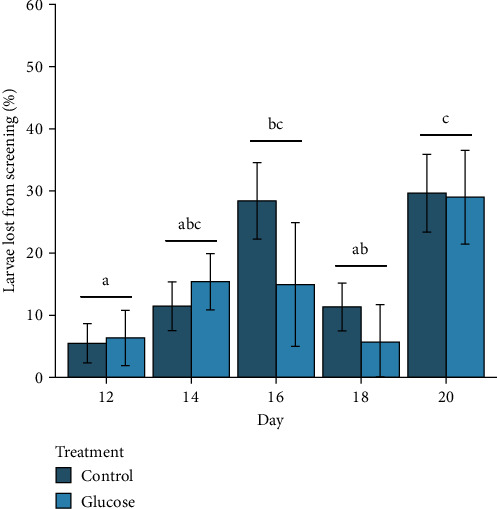
The mean proportion of larvae lost during screening at each of the five screening events during the larval rearing phase of the experiment, which included a control and glucose feeding treatment. Pairs of means for each screening event, regardless of treatment, with different letters are different (*p* < 0.05).

The mean proportion of larvae lost across the five screening events ranged from 5.7% (±1.8 SE) to 30.3% (±1.8 SE) between the two treatments. There was no significant interaction between treatment and day (*F*_(4,50)_ = 0.50, *P* = 0.73) or a significant fixed effect of treatment (*F*_(1,50)_ = 0.16, *p* = 0.69) on losses of larvae during screening over the 10-day larval rearing period (Figure 2). However, the effect of day on larval losses from screening was significant (*F*_(4,50)_ = 6.1, *p* = 0.001), with larval losses from screening Day 20 being higher than Days 12 and 18 (Figure 2). Larvae losses from screening on Day 16 were also greater than losses on Day 12.

### 3.3. Microalgae Cell Consumption

**Figure 3:**
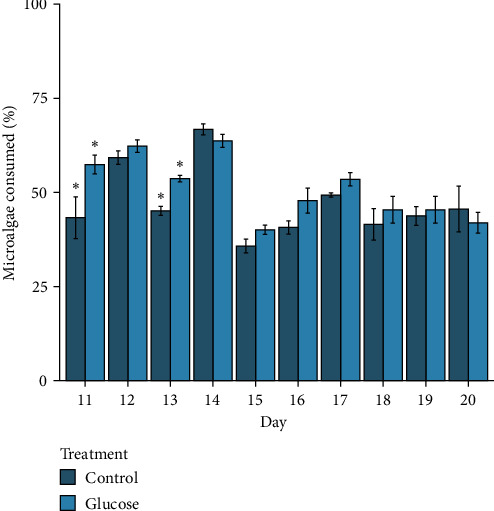
The mean percentage of microalgae cells consumed by larvae in control and glucose-feeding treatments over the 10-day period of larval rearing for the experiment. Means with different asterisks represent days where mean microalgae consumption by larvae was different between treatments (*p* < 0.05).

The mean proportion of phytoplankton consumed by larvae over the 10-day experimental period ranged from 35.8% (±1.8 SE) to 66.7% (±1.5 SE) between the two treatments. There were differences in the mean percentage of microalgae consumed due to the interaction between the treatments and days (*F*_(9,70)_ = 2.1, *p* = 0.04, Figure 3), with spat in the glucose treatment consuming a greater percentage of microalgae on Day 11 (*p* = 0.001) and 13 (*p* = 0.01).

### 3.4. Settlement Success

**Figure 4:**
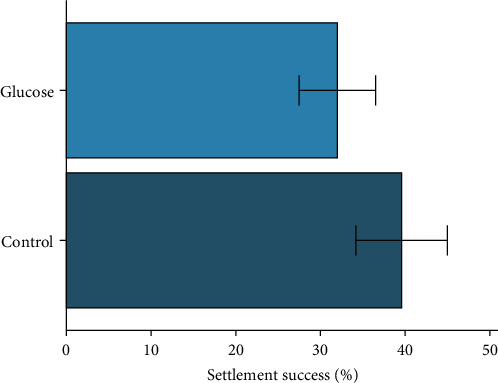
The mean percentage of larval settlement success in the glucose and control treatments after the 5-day settlement phase of the experiment.

The mean settlement success for pediveligers at the end of the 5-day settlement phase of the experiment ranged from 16.5% to 53.3% for individual tanks. The mean settlement success for larvae was 32.0% (±4.5 SE) in the glucose treatment and 39.6% (±5.4 SE) in the control (Figure 4). There was no significant difference in mean settlement success of larvae between the control and glucose treatments (*t*_(9.7)_ = 1.01, *p* = 0.31).

### 3.5. Analysis of Stable Isotopic Composition (*δ*^13^C and *δ*^15^N)

**Figure 5:**
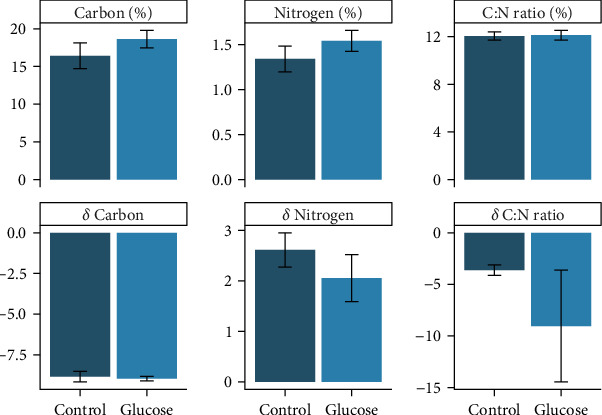
The mean percentage and delta value (‰) of carbon and nitrogen, and corresponding C:N ratios in mussel plantigrades for the control and glucose feeding treatments.

The carbon content of the plantigrades at the end of the 5-day settlement phase of the experiment ranged from 10.6% to 21.2% for individual tanks, and the nitrogen content ranged from 0.8% to 1.9%. The mean ^13^C% of the plantigrades was 16.4% (±1.7 SE) in the control and 18.6% (±1.4 SE) in the glucose treatment, with the means not being different (*t*_(7.0)_ = 1.10, *p* = 0.32) (Figure 5). The mean ^15^N% of plantigrades in the control treatment was 1.3% (±0.1 SE) and 1.5% (±0.12 SE) in the glucose treatment, with there being no difference between the two treatments (*t*_(7.6)_ = −1.08, *p* = 0.31, Figure 5). Further, there was no difference in the ratio of carbon to nitrogen content of the larvae (i.e., C:N) between the two treatments (*t*_(7.8)_ = −0.12, *p* = 0.91) with *t* the mean C:N for the control being 11.7 (±0.3 SE), while the glucose treatment was 12.4 (±0.4 SE).

Additionally, there was no difference in the mean delta *δ*^15^N (*t*_(7.3)_ = 0.97, *p* = 0.36) or mean *δ*^13^C (*t*_(5.4)_ = 0.34, *p* = 0.75) between the control and glucose treatments. The mean delta *δ*^15^N for spat in the control treatment was 2.6 ‰ (±0.3 SE) and 2.1‰ (±0.5 SE) in the glucose treatment, while the mean *δ*^13^C content of spat was –8.8‰ (±0.32 SE) in the control and –9.0‰ (±0.14 SE) in the glucose treatment. Also, there was no difference in the *δ*C:N ratio between the two treatments (*t*_(4.1)_ = 1.00, *p* = 0.38). The carbon to nitrogen ratio (C:N) for the control was –3.6 (±0.51 SE), while the *δ*C:N ratio for the glucose treatment was –9.0 (±5.42 SE).

## 4. Discussion

The high cost of producing live microalgae feed for bivalve larvae is a constraint on the operating efficiency of bivalve hatcheries worldwide [42]. Improvements in the delivery of more cost-effective alternative feeds for rearing bivalve larvae have the potential to play a key role in lowering overall operational costs for hatcheries. Providing bivalve larvae with soluble nutrients offers a promise as a cost-effective and readily available alternative feed, as bivalve larvae can uptake dissolved sugars, amino acids and vitamins from seawater [24–28]. Previous studies have demonstrated that sucrose and glucose can enhance the growth of Greenshell mussel spat when provided as a supplement to live microalgae [38, 39]. This study aimed to assess whether dissolved glucose could also be used to partially replace live microalgae in larval rearing of Greenshell mussels, with the potential for reducing live feed costs for hatchery operation.

Substituting live microalgae with dissolved glucose for 2 h per day between Day 10 and 20 of larval rearing had no effect on the larval size or daily growth of Greenshell mussel larvae. The mean prodissoconch length of larvae after 20 days in this study was significantly larger than those reported in previous studies, where the mean prodissoconch length after 20 days was less than 215 μm [8, 36]. In contrast, the larvae in this study reach mean lengths of 247 μm (control) and 241 μm (glucose treatment). These differences in larval size may be attributed to the benefits of using a diet containing three species of microalgae in the current study, compared to only two species of microalgae that had previously been used. This finding also highlights the high nutritional value of the microalgae diet provided in this study, which allowed larvae to reach the pediveliger stage earlier.

The uptake of DOM, including carbohydrates and amino acids, has been previously documented in the larvae of a range of bivalve species. Both larval oysters (*Magallan gigas* and *Ostra edulis*) and mussels (*Mytilus edulis*) have demonstrated the uptake of labelled glycine, which appeared in the velum of the larvae within 1 min of exposure and subsequently distributed throughout their tissues and organs [27]. Similarly, larvae of the Pacific oyster have been shown to uptake glucose from seawater when it is available at concentrations as low as 900 nM [28]. The concentration of glucose in this study (55.5 μM) was much higher, more than sufficient for uptake during the 2 h exposure. Greenshell mussel spat can absorb dissolved glucose from seawater over a 2 h period and use this to fuel growth when used as a supplement to live microalgae [38]. Although glucose uptake occurs through different structures in larvae and spat (with larvae using the velum and spat using gills), bivalves at both these stages of juvenile development can uptake and metabolise the available DOM [27].

While carbohydrates are recognised as the primary energy source for Greenshell mussel spat [10, 43–46], the role of carbohydrates in the nutritional requirements of Greenshell mussel larvae is not well understood. The microalgae used in this study (*C. calcitrans*, *T. lutea* and *C. muelleri*) vary in proximate composition but are generally higher in protein and lipid, with carbohydrate accounting for less than 15% of their ash-free dry weight [47–49]. Most carbohydrates in these species of microalgae are present as polysaccharides, which are complex molecules composed of multiple sugar units linked together [15]. Substituting part of this diverse carbohydrate profile, as well as lipid and protein, with only dissolved glucose may introduce nutritional limitations, particularly as proteins and lipids are believed to be key for larval development [14, 31, 34, 50]. Despite the likely reduction in protein and lipid intake resulting from reduced overall microalgae consumption in this current study, no significant differences in growth or nutritional status, as measured by carbon and nitrogen content, were observed between treatments. This suggests that glucose may have supported larval growth indirectly, potentially by sparing the catabolism of protein and lipids for energy [44, 46].

As larvae grow in size, their nutritional requirements generally increase, resulting in the increasing consumption of microalgae during development, with a sharp decline at the onset of metamorphosis when the larval feeding structures are lost [51]. In this study, the rates of consumption of microalgae did not increase consistently as larvae developed, with both the control and glucose treatments consuming between 35.8% and 66.9% of the microalgae provided throughout the 10-day experiment. Feeding by Greenshell mussel larvae 8 days after fertilisation is thought to be optimal at 60 microalgae cells μL^−1^ [8]. Although microalgae cell concentrations and rearing techniques were identical (i.e., 60 cells μL^−1^), spat were larger after 20 days of larval rearing compared to those reported in Ragg et al. [8], suggesting that tri-specific diets that include *C. muelleri* are of greater nutritional value to larvae. Furthermore, the broodstock used in this experiment may have superior genetics, contributing to faster larval development [52].

Settlement success, which is crucial for spat yield from a hatchery, is heavily influenced by diet quality, as adequate nutritional reserves are required for larvae to survive the non-feeding period during metamorphosis [13, 30]. Settlement success (i.e., spat yield) varies widely among bivalve species and studies, even those using mixed-species microalgal diets, with reported yields ranging from 25% to 72% [51, 53, 54]. This study observed a mean settlement success of 32% to 40%, which aligns with expectations with larvae fed a high-quality diet. Replacing microalgae with dissolved glucose for 2 h each day did not alter settlement success, indicating that dissolved glucose could be incorporated into a dietary regime for larval rearing to reduce reliance on cultured live microalgae.

Stable carbon and nitrogen isotope analyses are commonly utilised in ecology to ascertain nutrient assimilation and the relative dietary contributions of various food sources [55, 56]. A shift in carbon isotopes in the glucose-replaced treatment would suggest that the larvae are incorporating a higher proportion of carbon in tissue from the glucose, potentially reflecting a change in metabolic pathways or nutritional condition [57]. Nitrogen isotopes, which are more directly tied to protein synthesis, may show less variability, but any differences could indicate shifts in nitrogen retention or protein metabolism related to the different feeding regimes [58]. In this study, there were no significant differences in the carbon, nitrogen, or C:N ratio of post-settlement spat tissues between the control and glucose treatments, indicating that the reduction in feeding of live microalgae and replacement with dissolved glucose for 2 h a day did not affect the nutritional status of the Greenshell mussel larvae.

Previous studies have estimated that microalgal production in bivalve hatcheries represents 20%–40% of total operating expenses [12, 13, 59]. Based on this range, replacing live microalgae requirements for 2 h/day with dissolved glucose could reduce total operational costs by 2%–3%. However, is it possible that the feeding regime used here in the control treatement supplied larvae with more microalgae than they required. Accounting for the total reduction in feed, including tank flushes, feed was reduced by 11% without any detectable effect. This would suggest potential savings of 3%–4% could be achieved by optimising existing microalgal feeding practice. Larger-scale experiments are now required to establish whether the comparable outcomes under reduced microalgae feeding were due to the nutritional contribution of dissolved glucose or whether the control regime provided excessive feed beyond the requirements of the larvae. Future commercial-scale experiments should include negative feed controls, as this will help establish whether the greatest cost savings are likely to come from refining microalgae use or from incorporating dissolved glucose as a partial substitute in hatchery feeding regimes.

## 5. Conclusion

The hatchery production of mussel spat is a more commercially effective source of juveniles compared to wild-caught. However, the development of cost-effective alternative feeds to live microalgae is essential for improving the efficiency and economic viability of hatchery-produced mussels. This experiment demonstrated that under experimental conditions, replacing the microalgae diet of Greenshell mussel larvae with dissolved glucose (10 μg mL^−1^) for 2 h daily over 10 days of larval rearing resulted in no differences in the growth, settlement success, screening losses, the percentage of microalgae consumed during feeding periods or the nutritional status of mussels. This study has highlighted the potential of dissolved glucose as potential feed in bivalve hatcheries, although further work is required to determine how dissolved nutrients, like glucose, can be used most effectively to maximise cost savings on commercial scales.

## Data Availability

The data are available upon reasonable request from the corresponding author.
